# Understanding false positives in control conditions: a simulation study of *post-hoc* testing in low-powered neuroimaging trials

**DOI:** 10.3389/fnimg.2025.1637148

**Published:** 2025-12-04

**Authors:** Ji Hyun Ko

**Affiliations:** 1 Department of Human Anatomy and Cell Science, Rady Faculty of Health Sciences, University of Manitoba, Winnipeg, MB, Canada; 2 PrairieNeuro Research Centre, Kleysen Institute for Advanced Medicine, Health Science Centre, Winnipeg, MB, Canada; 3 Graduate Program in Biomedical Engineering, Price Faculty of Engineering, University of Manitoba, Winnipeg, MB, Canada

**Keywords:** interaction effects, simulation, false positives, control conditions, test–retest, Berkson’s paradox

## Abstract

Randomized controlled trials (RCTs) are essential for evaluating treatment efficacy, typically comparing active interventions to control conditions. In situations where blinding is impractical—such as in psychological therapies or physical rehabilitation—waitlist controls are often used to account for natural symptom progression and test–retest variability. This study examines the biases introduced by *post-hoc* analyses under conditions of low statistical power, particularly in neuroimaging research. Through large-scale simulations involving 100 million datasets with varying sample sizes, treatment effects, and test–retest variability, the study demonstrates that the common practice of conducting *post-hoc* tests only on brain regions showing significant interaction effects can substantially increase the false positive rate in the control condition. These findings underscore the relevance of Berkson’s paradox in interpreting unexpected control group outcomes and caution against overinterpreting such results. A complementary neuroimaging simulation reinforces these conclusions, emphasizing the need for critical scrutiny when evaluating significant effects in control groups. Overall, this work challenges conventional *post-hoc* testing strategies and advocates for a more nuanced and statistically informed interpretation of results, especially in studies with limited power.

## 1 Introduction

Randomized controlled trials (RCTs) are the gold standard for evaluating treatment efficacy, typically involving comparisons between an active intervention and a control condition. In many cases, control participants receive a placebo; however, when withholding treatment is ethically problematic, a gold-standard intervention may be used instead. In trials where blinding is not feasible—such as those involving psychological or physical therapies—waitlist control designs are commonly employed to account for natural disease progression and to estimate test–retest variability. A central statistical objective in these designs is to detect a significant 2 × 2 interaction between time and treatment on outcome measures (Hariton and Locascio, 2018), followed by *post-hoc* pairwise comparisons to interpret the directionality of effects (Garofalo et al., 2022). Ideally, these analyses reveal a significant improvement in the intervention group with no change in the control group. However, real-world data often yield unexpected patterns, such as X-shaped interactions or significant changes in waitlisted participants over short intervals. These outcomes are frequently dismissed as false positives attributed to procedural bias, raising concerns about the validity of the observed interaction effects.

Rigorous corrections for multiple comparisons are routinely applied to control false positives in neuroimaging analyses (Friston et al., 1995; Chumbley et al., 2010). However, these corrections are typically confined to the initial interaction effects, thereby narrowing the subset of brain regions subjected to subsequent *post-hoc* analyses. In addition to the well-documented inflation of effect sizes and exaggerated *p*-values in true positive cases (Kriegeskorte et al., 2009; Vul et al., 2009), this selective conditioning introduces a structural bias that increases susceptibility to Berkson’s paradox, wherein conditioning on a significant interaction can induce spurious associations in follow-up comparisons. When combined with the small sample sizes common in neuroimaging—due to high imaging costs—this bias can lead to the identification of clusters where apparent effects are driven by changes in the control group, whether stemming from placebo responses, natural variability, or random noise. In this study, we use large-scale computer simulations to systematically investigate this issue, demonstrating how the standard practice of performing *post-hoc* tests only on regions identified through interaction analyses can inflate false positive rates in the control condition. These findings highlight the importance of recognizing the statistical artifacts introduced by selective testing and underscore the need for more robust analytical strategies and cautious interpretation of control group effects in neuroimaging trials.

## 2 Methods

### 2.1 Computer simulation

All simulations were conducted using MATLAB R2023a (MathWorks Inc., Natick, MA). For each simulation, two groups—active and control—were generated using normally distributed random numbers to represent pre-condition measurements. Sample sizes varied from 5 to 50 per group, in increments of 5. Post-condition values were defined as the sum of the pre-condition and additional normally distributed noise, scaled by weights ranging from 0.1 to 1.0 (incremented by 0.1) to simulate varying levels of test–retest variability. In the active group, treatment effects were introduced by adding fixed values to the post-condition, with effect sizes ranging from 0.1 to 1.0 (incremented by 0.1). Each parameter combination was simulated 100,000 times. Of these, 10% (10,000 simulations) included a true treatment effect, while the remaining 90% did not—mimicking a scenario in which only a subset of brain regions (e.g., 10%) are genuinely affected by the intervention.

### 2.2 Statistical analysis

Interaction effects were assessed using the fitrm function in MATLAB, which fits repeated-measures models to evaluate the 2 × 2 interaction between time and treatment. Resulting *p*-values were corrected for multiple comparisons using the false discovery rate (FDR) procedure, yielding q-values—defined as the minimum FDR at which a given test result is considered significant (Benjamini and Hochberg, 1995). The false positive rate for interaction effects was estimated as the proportion of significant results (*q* < 0.05) among simulations without a true treatment effect (i.e., 90,000 cases). Sensitivity was defined as the proportion of significant results among simulations with a true treatment effect (i.e., 10,000 cases). To quantify the influence of experimental parameters—sample size, test–retest variability, and treatment effect size—on sensitivity, multiple linear regression was performed. For each significant interaction effect, a paired *t*-test was conducted within the control group to assess the likelihood of spurious declines, serving as a proxy for false positives in *post-hoc* comparisons. Finally, curve fitting was applied using MATLAB’s curveFitter function to model the relationship between interaction test sensitivity (i.e., statistical power) and the rate of false positive findings within the control group.

Additionally, to assess whether low statistical power systematically biases the estimation of true effect sizes, we calculated the difference between the estimated effect size (i.e., the beta coefficient) and the true effect size (i.e., the simulated treatment effect) for each true positive case—defined as instances where an effect was both simulated and detected via the interaction effect test. These differences were then averaged within each simulation setting. The same statistical analyses described above were applied, with the dependent variable now being the change in effect size (∆effect size) observed in true positive cases.

### 2.3 Neuroimaging sample used for simulation

To simulate neuroimaging data from individuals undergoing treatment, we utilized real data from healthy participants who were part of a previous neuroimaging study conducted by our group. These individuals, free from any neurological or psychiatric disorders, were originally recruited as the control group in a clinical trial investigating the effects of cognitive processing therapy in patients with posttraumatic stress disorder (PTSD). Due to ethical constraints preventing the withholding of active treatment from individuals with PTSD, the control group (*n* = 24) underwent the same clinical assessments and neuroimaging protocols as the treatment group. Details of the original study will be reported elsewhere (Wright et al., under review). Each participant completed two MRI sessions—T1-weighted structural imaging and resting-state functional MRI (fMRI)—spaced 4 months apart.

All procedures were approved by the University of Manitoba Biomedical Research Ethics Board, and written informed consent was obtained from all participants. The study was conducted in accordance with institutional guidelines and relevant regulations.

MRI data were acquired at the Health Sciences Centre in Winnipeg, Manitoba, using a 3 T Siemens/IMRIS MR system equipped with a 12-channel head coil. During the 11-min resting-state fMRI scan, participants were instructed to keep their eyes open, allow their minds to wander, and avoid falling asleep. Imaging parameters for the resting-state scan were: repetition time (TR) = 2000 ms, echo time (TE) = 28 ms, flip angle = 77°, slice thickness = 4 mm, voxel size = 3.4 × 3.4 × 4.0 mm, and field of view (FOV) = 220 mm. High-resolution structural images were acquired using a 3D T1-weighted MPRAGE sequence with the following parameters: inversion time (TI) = 900 ms, TR = 2,300 ms, TE = 3.02 ms, flip angle = 9°, slice thickness = 1 mm (240 slices), voxel size = 1.0 × 1.0 × 1.0 mm, and FOV = 220 mm.

### 2.4 Estimating intrinsic connectivity from resting-state fMRI

All preprocessing and image analyses were conducted using SPM12 (Friston, 2007) and the CONN toolbox v20b (Whitfield-Gabrieli and Nieto-Castanon, 2012) in MATLAB R2023a. Preprocessing followed CONN’s default pipeline, including realignment with susceptibility distortion correction, slice-timing correction, outlier detection, segmentation, normalization to MNI space, and spatial smoothing. Functional images were realigned to the first scan of the first session using SPM’s realign and unwarp procedure. Slice timing was corrected, and outlier volumes were flagged based on framewise displacement (>0.9 mm) or global BOLD signal changes (>5 SD). Functional and anatomical images were segmented and normalized using SPM’s direct normalization with default tissue probability maps. Spatial smoothing was applied using an 8 mm FWHM Gaussian kernel.

Following preprocessing, standard denoising was performed using CONN’s linear regression pipeline, including white matter and CSF signals, motion parameters, scrubbing, and quality control time series as confounds. Intrinsic connectivity (IC) maps were then computed for each subject, defined as the root mean square of correlation coefficients between each voxel’s time series and all other voxels in the brain (Martuzzi et al., 2011), yielding voxel-wise maps of global functional connectivity.

### 2.5 Simulating treatment effects in neuroimaging data

**Figure 1:**
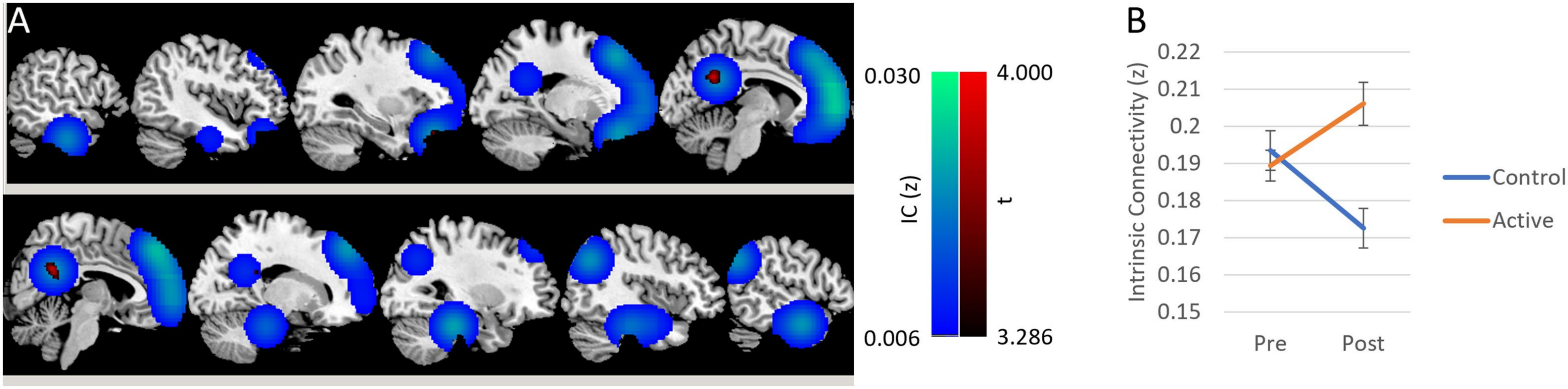
Simulated neuroimaging study with low sensitivity interaction effects. **(A)** Simulated images of treatment effect is overlaid in blue on a template MRI. Statistical parametric mapping of interaction effects in intrinsic connectivity (IC) between group and time is overlaid in red. False discovery rate (FDR)-correction at cluster level (*q* < 0.05) was used with uncorrected peak-level threshold (*p* < 0.001). Only one cluster was found to be significant (*q* = 0.027, *k* = 425) centered in the posterior cingulate cortex (*x* = 4, *y* = −54, *z* = 28). **(B)** The mean IC within the cluster was estimated from each individual, and repeated measures general linear model analysis was performed. The significant interaction effect was replicated (*F*(1, 22) = 17.1, *p* < 0.001). The *post-hoc* Bonferroni test showed a significant increase in the active group (*p* = 0.016), while significant reduction in the control group was also observed (*p* = 0.016).

To simulate a “treatment” effect, 11 regions of interest (ROIs) were defined based on established default mode network coordinates (Lin et al., 2017). Each ROI was initialized with a voxel intensity of 100 at the target coordinate, smoothed using a Gaussian kernel (FWHM = 7 mm), and thresholded at >0.005 to generate spatially distributed activation patterns. These simulated treatment effect images (Figure 1A, blue) were then added to the post-treatment scans of 12 randomly selected healthy participants.

Group-by-time interaction effects were assessed using SPM12, applying a peak-level threshold of *p* < 0.001 (uncorrected) and a cluster-level threshold of *q* < 0.05 (FDR-corrected). Mean IC values were extracted from the resulting significant clusters and analyzed using repeated-measures general linear modeling in IBM SPSS Statistics (v27; IBM Corp., Armonk, NY). *Post-hoc* Bonferroni-corrected tests were conducted to evaluate within-group changes over time.

## 3 Results

**Figure 2:**
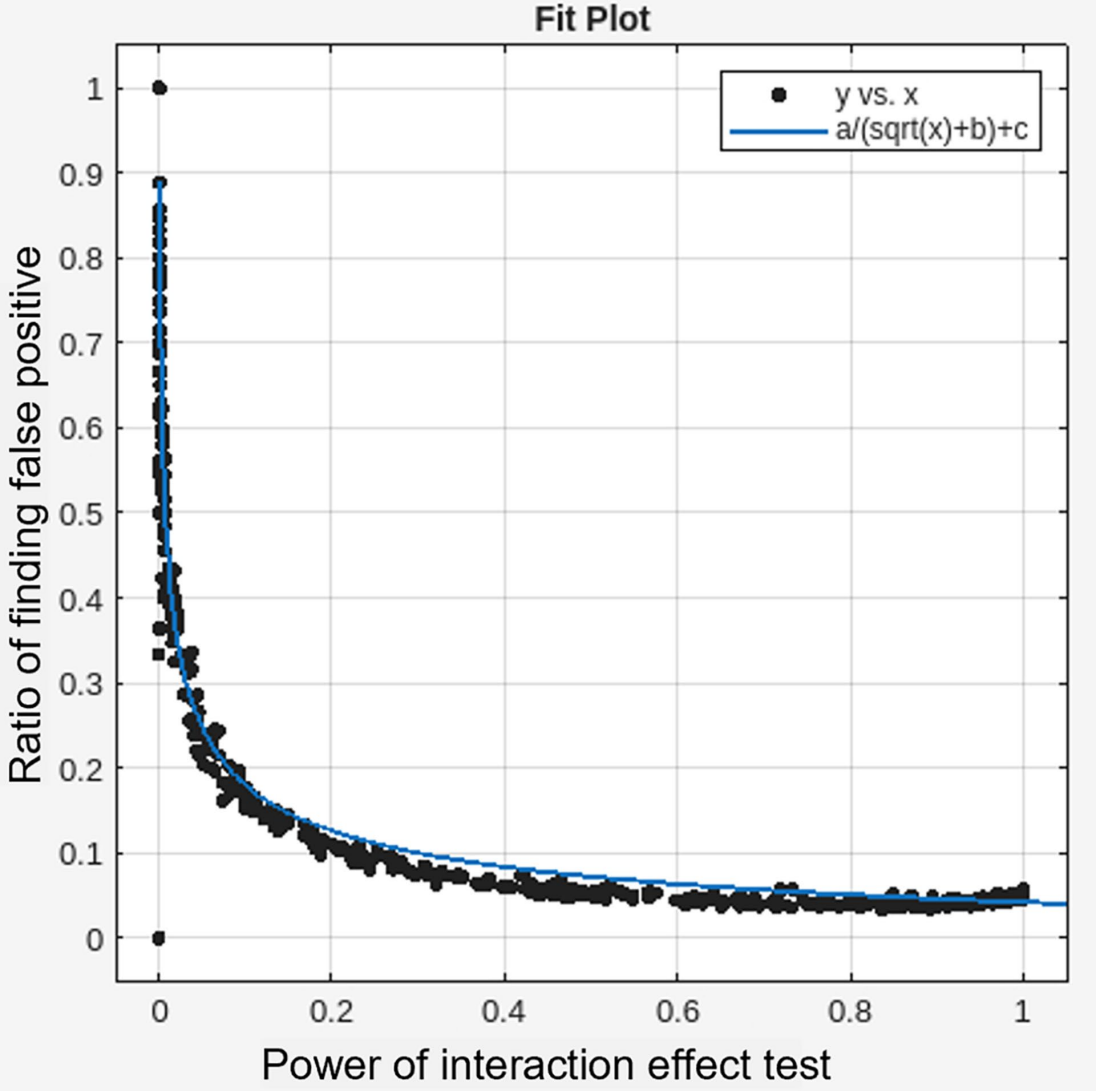
Inversely proportional relationship between the sensitivity of interaction effects (x-axis) and ratio of finding false positive effects in a control condition (y-axis). Curves are fitted by a/(sqrt(x) + b) + c. a = 0.0883; b = 0.0850; c = −0.0408; Adjusted *R*^2^ = 0.9097.

**Figure 3:**
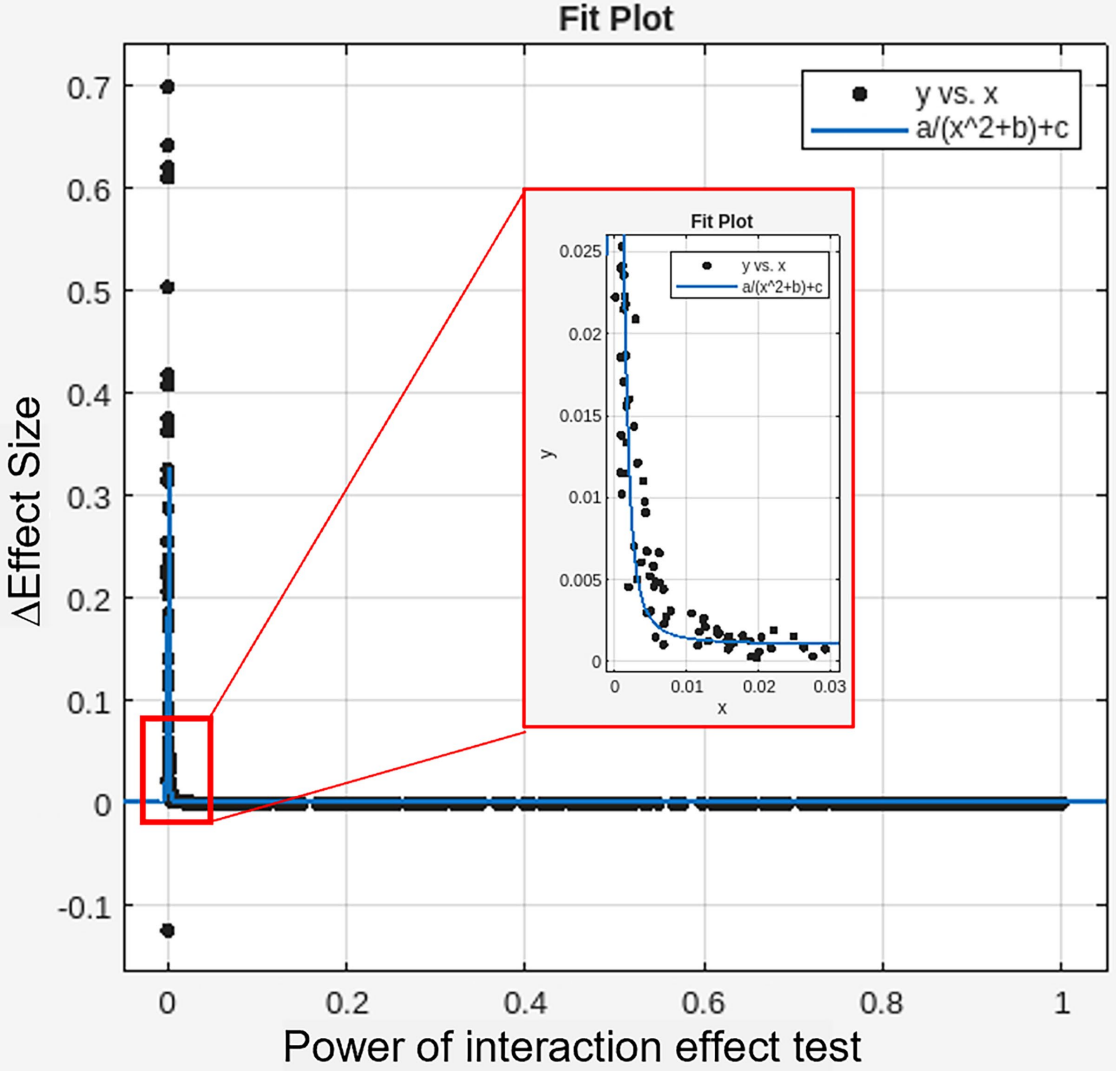
Inversely proportional relationship between the sensitivity of Interaction effects (x-axis) and inflated effect sizes (estimated minus simulated effect sizes) in true positive findings (y-axis). Curves are fitted by a/(x^2 + b) + c. a = 1.986e-08; b = 4.525e-08; c = 4.770e-04; Adjusted *R^2^* = 0.7404.

A total of 100,000,000 simulated datasets were generated to evaluate 2 × 2 interaction effects (group × time), varying sample sizes (*n* = 5–50 per group), treatment effect sizes (*z* = 0.1–1.0), and test–retest variability (*z* = 0.1–1.0). In each simulation, 10% of the active group datasets included a true treatment effect. Across all simulations, the false positive rate for interaction effects remained below 0.6%. As expected, the statistical power to correctly identify significant interaction effects varied widely across conditions (0–100%) and was significantly associated with treatment effect size (*t*(996) = 36.4, *p* < 0.001), test–retest variability (*t*(996) = −33.4, *p* < 0.001), and sample size (*t*(996) = 22.4, *p* < 0.001) (Table 1). Among simulations with true interaction effects, false positives in the control group ranged from 0 to 100%, and this rate was strongly associated with the overall sensitivity of the interaction test (adjusted *R*^2^ = 0.9097), the relationship of which was best explained by a root squared inverse variation function (Figure 2). Among true positive cases, effect sizes were systemically inflated especially when the statistical power was below 5%, and the relationship of which was best explained by reciprocal quadratic function (Figure 3).

**Table 1:** Influence of simulation parameters to sensitivity (statistical power).

	Simulated range	Beta	*t*	*p*
Treatment effect size	0.1–1.0	0.899	36.4	<0.001
Test–retest variability	0.1–1.0	−0.826	−33.4	<0.001
Sample size	5–50	0.011	22.4	<0.001

Adjusted *R*^2^ = 0.746.

To simulate a more realistic neuroimaging scenario, IC maps derived from resting-state fMRI data of 24 healthy individuals (age: 33.8 ± 13.8 years; 10 males, 14 females) were used. These participants, who received no intervention, were scanned twice over a four-month interval as part of a clinical trial (ClinicalTrials.gov ID: NCT03229915). Simulated treatment effects were introduced into 11 ROIs based on default mode network coordinates (Lin et al., 2017) (Figure 1A). A significant group × time interaction was detected in a single cluster located in the posterior cingulate cortex (PCC; *q* = 0.027, FDR-corrected at the cluster level; *p* < 0.001 uncorrected at the peak level; Figure 1A). Mean IC values extracted from this cluster were analyzed using a 2 × 2 repeated-measures general linear model, confirming a significant interaction effect (*F*(1, 22) = 17.1, *p* < 0.001; Figure 1B). *Post hoc* Bonferroni-corrected comparisons revealed a significant increase in IC in the simulated treatment group (*p* = 0.016) and a significant decrease in the control group (*p* = 0.016), consistent with the simulation’s predicted bias.

## 4 Discussion

It is a widely accepted practice in clinical and neuroimaging research to perform *post-hoc* pairwise comparisons only when omnibus interaction effects are statistically significant. However, low statistical power in detecting interaction effects can systematically increase the likelihood of observing false positive outcomes in control conditions as well during *post-hoc* analyses. Specifically in our simulation study, when the power of the interaction test was 20, 11.4% of the resulting significant findings showed false positives in the control group. When the power dropped to 5%, this rate increased to 22.0%. Notably, when the power approached 80%—a common benchmark for statistical power—the false positive rate in the control group was reduced to approximately 5%. These results underscore the critical importance of conducting high-powered studies to minimize interpretive errors.

While this relationship may seem intuitive, it is often overlooked in practice. In neuroimaging, researchers frequently dismiss significant interaction effects if unexpected changes are also observed in the control group, assuming these to be artifacts or noise. However, our findings suggest that such outcomes may instead reflect a statistical artifact rooted in Berkson’s paradox. In this context, conditioning on a significant interaction effect—especially when only a small number of regions survive correction—can induce spurious associations in *post-hoc* comparisons. That is, selecting regions based on their interaction significance inherently biases the sample, increasing the likelihood of detecting false positives in the control group, even when no true change exists.

This phenomenon was clearly illustrated in our neuroimaging simulation. Although treatment effects were introduced in 11 default mode network regions, only one region—the PCC—was identified as significant. Importantly, this region also showed a significant decrease in IC in the control group. However, when the control group was analyzed independently using a whole-brain paired t-test with the same statistical threshold, no significant clusters were detected. This suggests that the observed control group effect was within the expected range of normal signal fluctuation and only appeared significant due to the biased selection of regions following an underpowered interaction analysis.

The problem of inflated significance in *post hoc* analyses has been prominently addressed by Kriegeskorte et al. (2009). Vul et al. (2009) famously coined the term “voodoo correlations” to describe the methodological pitfalls that can lead to overstated brain-behavior relationships. In response, a range of strategies has been proposed to mitigate false positives. These include the development of statistical techniques specifically designed for neuroimaging data (Lazar, 2009), the use of model-based predictive frameworks such as multivariate analysis (Cremers et al., 2017), and the adoption of pre-specified, transparent contrast definitions (Granziol et al., 2025). Validation through independent samples within the same ROI, or via cross-validation, can further support the robustness of findings—though these methods often necessitate larger sample sizes. While these approaches are primarily aimed at reducing false positives in active conditions, the underlying principles—such as rigorous statistical planning and the avoidance of circular analysis—are equally critical for minimizing false positives in control conditions.

In conclusion, this study highlights a critical vulnerability in standard neuroimaging analysis workflows: the inflation of false positives in control groups due to low statistical power and selective *post-hoc* testing. These findings advocate for more cautious interpretation of control group outcomes and emphasize the importance of improving statistical power—through larger sample sizes, reduced test–retest variability, or stronger treatment effects—to enhance the reliability of interaction effect analyses. Notably, when statistical power is sufficient to detect even one-third of true effects, the false positive rate in control conditions drops below 8.6%, reinforcing the value of well-powered study designs.

## Data Availability

The original contributions presented in the study are included in the article/supplementary material, further inquiries can be directed to the corresponding author.
